# Stepwise Retrieval of a Broken Pituitary Disc Rongeur

**DOI:** 10.7759/cureus.8561

**Published:** 2020-06-11

**Authors:** Richard P Menger, Zachary Lazzari, Anil Nanda

**Affiliations:** 1 Neurosurgery and Political Science, University of South Alabama, Mobile, USA; 2 Medicine, University of South Alabama College of Medicine, Mobile, USA; 3 Neurosurgery, Rutgers-Robert Wood Johnson Medical School and University Hospital, New Brunswick, USA

**Keywords:** complications, pituitary disc rongeur, discectomy, microdiscectomy

## Abstract

We report a case of a patient undergoing open microdiscectomy at L5-S1, wherein the distal tip of a pituitary rongeur became dislodged within the disc space. Complication management and avoidance of anterior advancement are paramount. A stepwise plan to retrieve the foreign body was entertained in a methodical fashion. Such device failure places the patient at greater risk of injury, extends operation time, and adds undue burden on the surgical team. This situation warrants discussing the complications from retained foreign bodies and measures taken for their removal.

## Introduction

Posterior open microdiscectomy is a widely used approach for lumbar disc pathologies similar in its goals as minimally invasive surgery [1,2]. While this approach possibly relies on greater muscle and fascial dissection, studies have not shown greater risk of complications when compared to alternative approaches (microendoscopic discectomy, percutaneous discectomy) [1]. This approach can, however, be complicated by rare, but serious iatrogenic vascular injury owing to pituitary disc rongeur protrusion through the anterior longitudinal ligament, and subsequently the retroperitoneal space [3]. Incidents of this nature are typically avoided by surgical care and intraoperative imaging, but these can also depend on the integrity of surgical instruments [4]. The pituitary disc rongeur allows for tissue removal in confined spaces, but its durability may diminish after many cycles of sterilization and use [4].

We present a case of a broken pituitary rongeur tip in the disc space and focus on avoiding further injury by delineating a method for retrieving foreign bodies from the disc space. Secondarily, we discuss risks associated with retaining foreign bodies and efforts to remove them from the disc space.

## Case presentation

**Figure 1:**
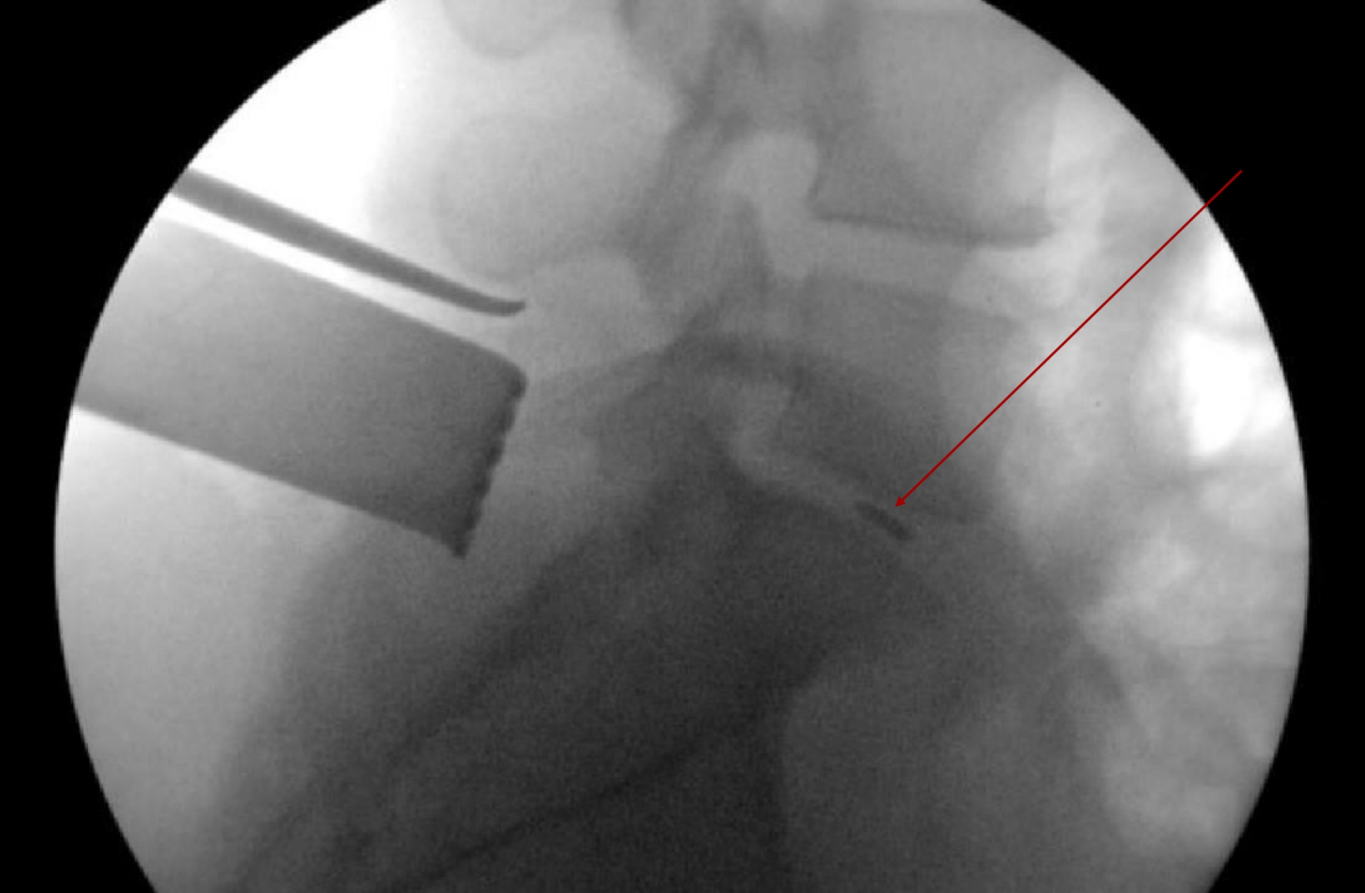
Lateral Radiograph of Broken Pituitary Disc Rongeur

A 37-year-old female underwent left-sided L5-S1 open microdiscectomy. Upon annulotomy, a pituitary disc rongeur was used to remove disc fragment material. During use it became apparent that the superior grasping tip of the rongeur was dislodged. Fluoroscopy confirmed the broken fragment to be in the disc space (Figure 1). 

**Figure 2:**
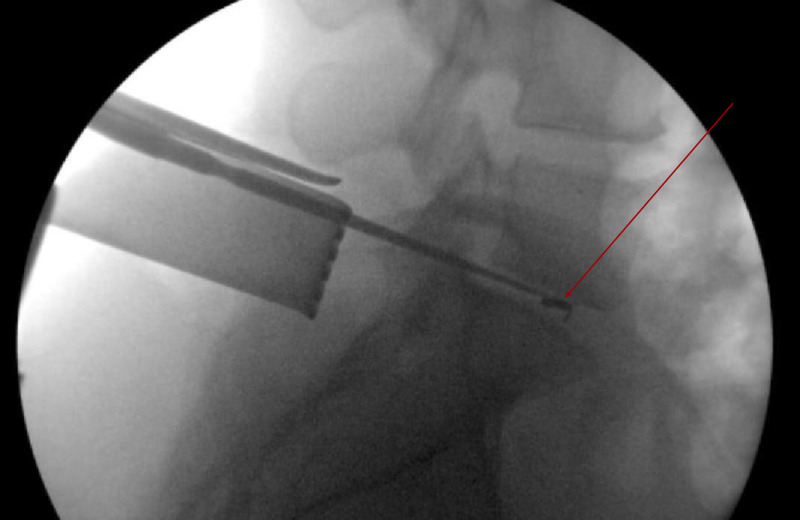
Lateral Radiograph of Blunt Nerve Hook Palpation of Broken Rongeur

**Figure 3:**
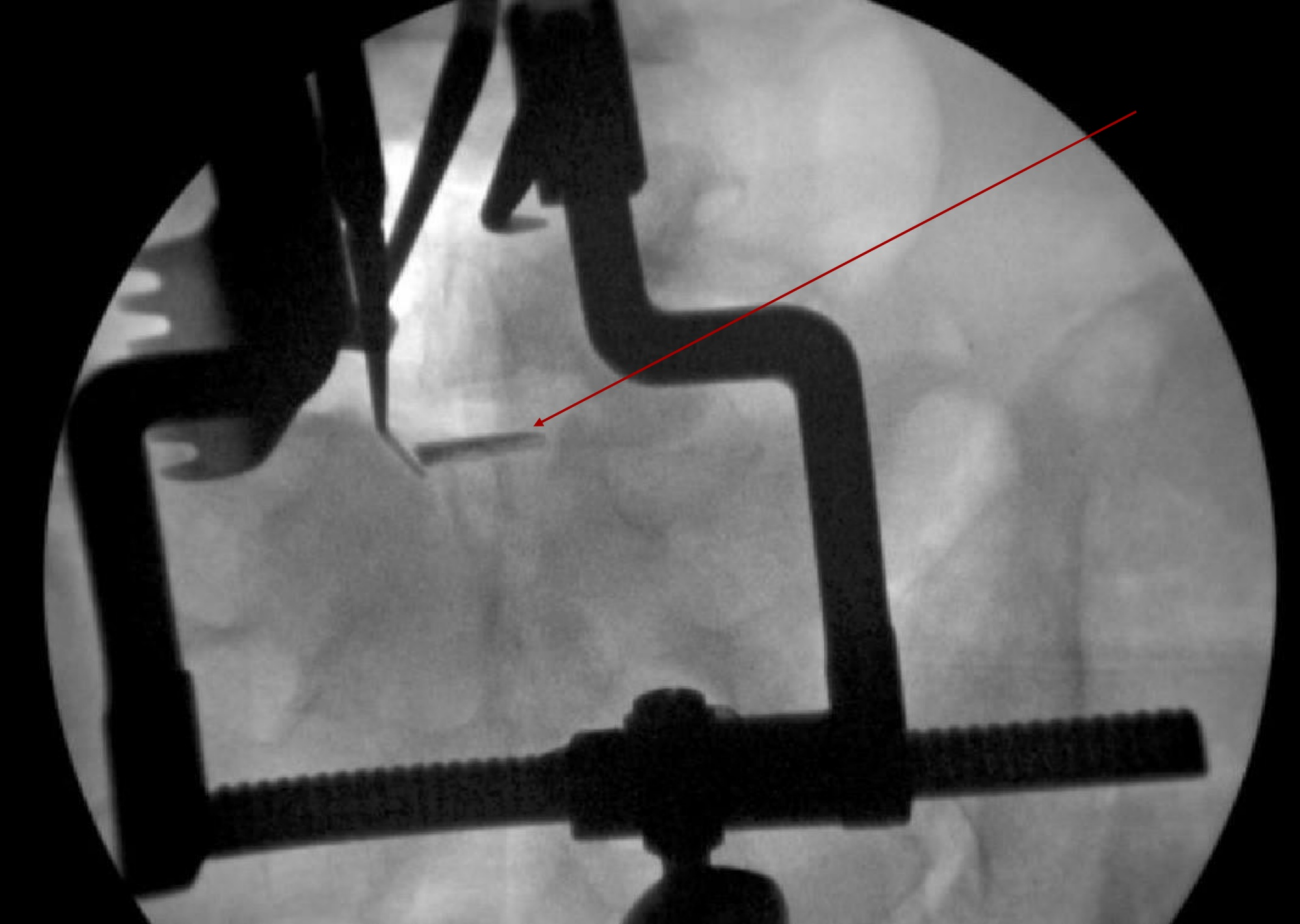
Anterior Posterior Radiograph of Attempted Removal of Broken Rongeur

Prior to further intervention, the resident and lead surgeon decided upon a stepwise approach to retrieving the piece; great care was taken not to further displace the artifact anteriorly. Increased superficial exposure of disc space was achieved. A magnet was then used to tease out the broken object. While changing the radiographic appearance, this was not sufficient to aid in extraction. Attempted removal of the foreign body was performed under live fluoroscopy with blunt nerve hook and Woodson instruments (Figures 2, 3). 

**Figure 4:**
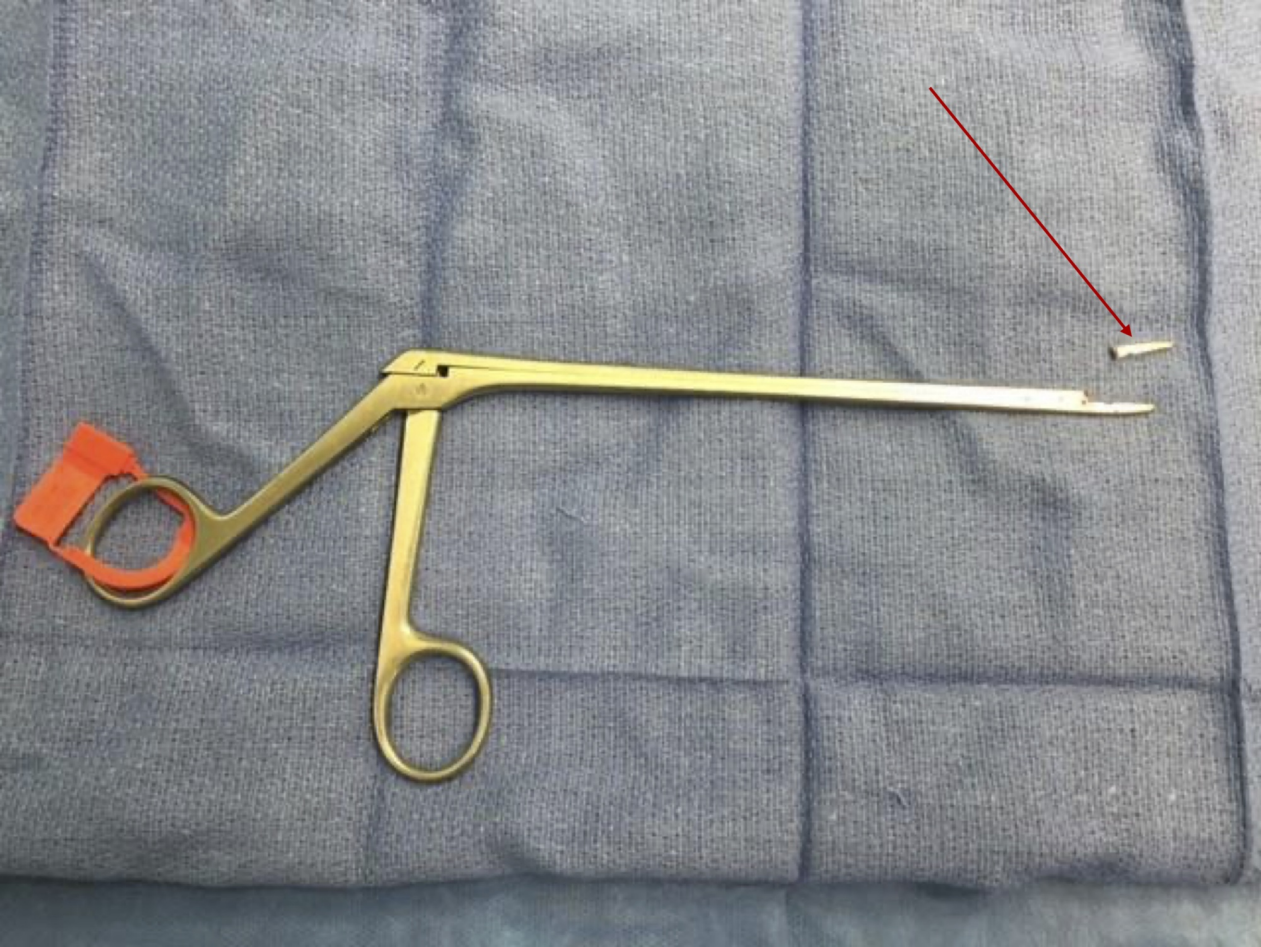
Successful Removal of Broken Rongeur Fragment and Original Instrument

After 1.5 hours, the decision was made to drill portions of the end plates to increase exposure. Eventually, the fragment was blindly removed by a pituitary disc rongeur (Figure 4). Direct visualization of the metal body in the disc space was never achieved. 

Other considerations in sequence considered the use of interbody shavers, conversion to open laminectomy for full inspection of the disc bilaterally, conversion to transforaminal interbody fusion with full discectomy, or finally performing an anterior approach to the disc space.

## Discussion

The focus is on proper operative instrumentation. This instrument failure increased operative time and put the patient at risk. Medical-legally this issue garners a discussion of the risks of a retained foreign body as compared to the risks of additional procedures or aggressive techniques.

A meta-analysis including 42 studies and 5,390 patients (of which 2,526 patients underwent open microdiscectomy) by Shriver et al. reveals open microdiscectomy complication rates of 3.1%, 1.3%, and 6.0% for surgical errors (including surgical instrument breakage), wound complications, and reoperation, respectively [1]. Instances of instrument failure increase the risk from all of these categories.

Sequelae from retained foreign bodies within the disc space may include granulomatous reactions, abscess formation, and/or compression of surrounding structures [5]. Cotton pads, towels, and sponges represent the more commonly retained foreign bodies, often presenting with infection in the early post-operative period [4,5]. However, metallic foreign objects have been shown to result in radiculopathy or other neurological consequences due to granulation tissue developing in confined regions of the spinal column [6-8]. Symptoms from retained foreign bodies may present acutely or delayed [4,5,7]. The risk of displacement into the spinal canal often compels surgeons to remove foreign objects rather than leave them [6].

Attempts to remove iatrogenic foreign bodies exacerbate the risks associated with spinal procedures, including nerve root injury, vascular complications, durotomy, infection, and reoperation [1]. Furthermore, attempts to remove a foreign object may incidentally advance it through the anterior longitudinal ligament [3]. Retroperitoneal vascular insult can ensue from anterior displacement without much evidence of bleeding in the surgical field of view due to elasticity of the anterior longitudinal ligament [3]. Anterior displacement of the rongeur tip can lead to bleeding, embolization, or other retroperitoneal insult [3]. Under certain conditions, the importance of removal may be diminished, but these decisions depend greatly on location and specifics for the case.

Both routes (removal or retention of foreign bodies) bear consequences and place greater demand on the surgical team and patient. The literature suggests that removal is largely favored over retention, though emphasizing the need for a methodological approach to safely and efficiently retrieve foreign objects from the disc space [4,7].

**Figure 5:**
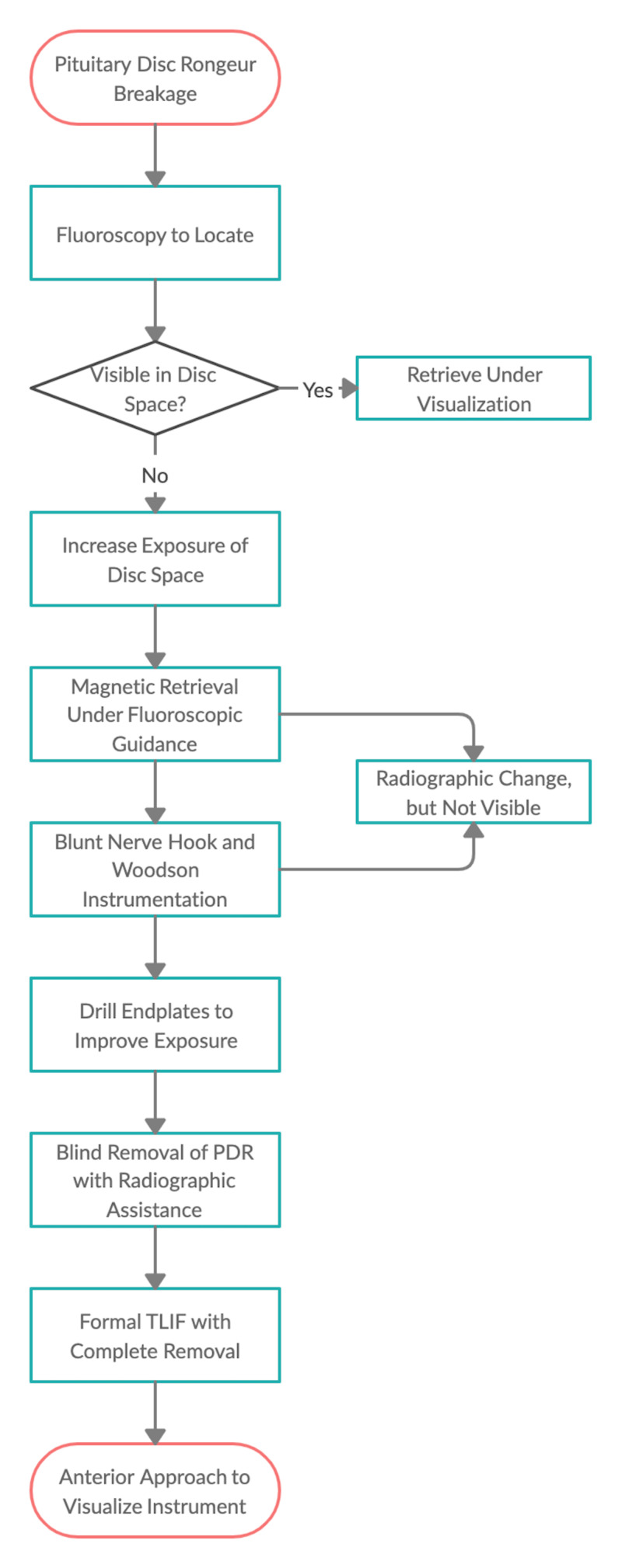
Methodology for Foreign Body Retrieval From the Disc Space PDR, pituitary disc rongeur; TLIF, transforaminal lumbar interbody fusion

Surgeons are exposed to high levels of stress by an array of factors, including equipment failure. Studies of stress responses in pilots have shown problem-focused coping strategies (dealing directly with stressor by formulating and executing a plan for resolution) contribute to higher levels of performance and lower error rates than other coping strategies [9]. Reviewing a stepwise approach to foreign body retrieval (Figure 5) may be beneficial in managing stress and reducing operative time should incidents of this nature occur.

## Conclusions

The methodology for foreign body retrieval from the disc space may present a safe, efficient guide to avoid further insult. There may be cases in which foreign body retention presents fewer risks than implementing aggressive measures for removal, but few discussions of the risks associated with either course have been offered in the literature. We have attempted to provide an overview above.
